# Identifying Organizational Stressors That Could Be a Source of Discomfort in Police Officers: A Thematic Review

**DOI:** 10.3390/ijerph19063720

**Published:** 2022-03-21

**Authors:** Daniela Acquadro Maran, Nicola Magnavita, Sergio Garbarino

**Affiliations:** 1 WOW—Work and Organisational Well-Being Research Group, Department of Psychology, Università di Torino, 10124 Torino, Italy; 2 Postgraduate School of Occupational Medicine, Università Cattolica del Sacro Cuore, 00168 Rome, Italy; nicolamagnavita@gmail.com; 3 Department of Woman/Child & Public Health, Fondazione Policlinico Universitario Agostino Gemelli IRCCS, 00168 Rome, Italy; 4 Department of Neuroscience, Rehabilitation, Ophthalmology, Genetics and Maternal/Child Sciences (DI-14 NOGMI), University of Genoa, 16132 Genoa, Italy; sgarbarino.neuro@gmail.com

**Keywords:** organizational support, leadership, organizational culture, bureaucracy

## Abstract

The aim of this paper is to highlight the organizational factors that might influence perceived discomfort in police officers. The studies included in the thematic review referred to specific factors, not the general terms “organizational stressors” or “workplace stressors”. It is important to emphasize this distinction because most studies use the general term “organizational stressor” (referring to context) to distinguish from “operational stressor” (referring to content, such as exposure to danger, threat, and trauma). For our purposes, we selected the studies that examined specific organizational factors. The results indicate that organizational social support, organizational culture, leadership, and bureaucracy are the organizational factors associated with police officers’ perceived discomfort. These organizational factors could have negative impacts on individuals, perceptions of stigma when contacting support services, anxiety and depressive symptoms, burnout, PTSD, and suicidal thoughts, among others.

## 1. Introduction

Police officers are professionals who are exposed to events such as contact with problematic situations both human and material, continued exposure to suffering, threats to their own safety and that of colleagues, the use of weapons, performance in emergency operations, and exposure to traumatic events such as death; these exposures result in distress [1,2,3,4,5,6]. For these reasons, police officers are considered employees with high levels of exposure to acute and chronic stressors [7,8] and lower levels of well-being compared with other similar occupations [9] and with the general population [10]. This professional group could experience consequences reflected in the risk of developing psychopathological disorders such as post-traumatic stress disorder (PTSD), acute stress disorder, and adaptation disorders; in addition, physical and psychosomatic consequences (sleep disorders, eating disorders, and cardiovascular disorders) should be reported [11,12,13,14,15]. Interestingly, as reported by Kerswell et al. [16], the sources of suffering are the same for operational and non-operational police officers, proving that direct contact is not the only source of suffering [10]. 

One reason for this seems to be the organizational factors rather than the occupational factors. As suggested by the scholars [17,18,19,20], organizational stressors seem to be the main cause of the stress process in police officers. Moreover, Garbarino et al. [21] found that depressive symptoms in police officers were related to organizational stressors rather than to individual stressors. Similarly, the results of a study by Suresh et al. [18] showed that stressors in police officers were related to organizational rather than other aspects of their work, such as job content. For example, Shane [22] indicated that perceptions of an increase in contextual stressors are related to a decrease in well-being and performance; high levels of organizational stress undermine police officers’ ability to better perform their operational duties [23]. As described by Shane [22] (2010), organizational stressors, also referred to as work context stressors, include the characteristics of the organization and the behaviors of the people in that organization that can generate stress. In police officers, these stressors can be perceived as oppressive, unnecessary, and inescapable. They include “lack of support, heavy workloads, interpersonal conflicts with colleagues and supervisors, inadequate resources, time pressures, and an overly bureaucratic organizational system that punishes and harshly manages employees” ([3], p. 2). Strijdom and Rothman [24] (2002) found that police officers with inadequate supervisory support, excessive administrative pressure, and low job satisfaction were at increased psychosocial risk. In a study by Pienaar et al. [25], police officers who perceived high levels of organizational stress were also more likely to use avoidant coping strategies when dealing with stressors. These, according to the authors, could lead to and/or exacerbate anxiety and depressive symptoms, burnout, PTSD, and suicidal ideation. As Chae and Boyle [26] noted, organizational stressors are the result of both the structural environment and the functional aspects of organizational life (see also [22,27,28]). In particular, the structural environment is related to organizational culture, which determines management style and lack of autonomy and influences interactions among officers. Functional aspects are related to shift work, irregular working hours, and consecutive working days, which can cause emotional stress [29,30]. 

In this review, we consider the potential stressors associated with organizational factors in policing. As argued, organizational stressors appear to be the primary cause of the stress process in police officers [17]. As described above, Shane [22] indicated that the perception of an increase in contextual stressors is related to a decrease in well-being and performance; high levels of organizational stress undermine the ability of police officers to better perform their operational tasks [23]. Shane [22] also pointed out that the effects of these types of stressors are relentless and unavoidable, making interventions a priority in police officer assistance programs. Organizational stress in police officers could be caused by the culture, conditions, rules, and procedures inherent in the organizational context in which they work. The interest in organizational factors that might affect police officers’ perceived discomfort lies in the possibility of intervention. While it is possible to change the organization specifically (e.g., by redesigning the work), individual factors are less susceptible to intervention: As Chae and Boyle [26] noted, people—including police officers—respond in different ways to traumatic events and life stressors, so “it should not be assumed that all individuals respond in the same way when confronted with psychological stress” (p. 108). The aim of this work was to identify the main organizational stressors that affect the well-being of police officers and lead to poor psychological outcomes (burnout, anxiety and depressive symptoms, maladaptive coping strategies, etc.) or, alternatively, to identify the aspects that can protect against stressful working conditions. Identifying these aspects could lead to a better understanding of the organizational stressors that can affect police officers’ well-being and how to intervene and prevent stress from an organizational perspective. We assumed that the good functioning of our psyche is a fundamental requirement for not only health but also for living a full and satisfying life, self-actualization, building good relationships, and asserting ourselves in the community [31]. The development of psychological well-being is essential not only for health (physical and mental) but also for social and professional lives.

## 2. Materials and Methods

A literature search was conducted in PsycINFO, SCOPUS, and Web of Science up to 11 January 2022. Three main terms were used in the search strategy: occupational stress and its different dimensions, “POLICE OFFICERS”, and keywords related to psychological well-being and health. Specifically, the search terms for PsycINFO were as follows: (“ORGANIZATIONAL STRESS” OR “JOB CONTEXT STRESS”) AND “PTSD” OR “POSTTRAUMATIC STRESS” OR “POST-TRAUMATIC STRESS” OR “BURNOUT” OR “DEPRESSIVE SYMPTOMS” OR “ANXIETY SYMPTOMS” OR “MALADAPTIVE COP-ING STRATEGY” OR “SUICIDAL THOUGHT” OR “SUICIDAL RISK”).

The same keywords were used for the literature search in the other databases accessed. In addition, a manual search was performed in the bibliography of the main papers. Papers were selected according to the concept of PICO, considering population, interventions, comparisons, and outcomes. The target population was police officers. The interventions were organizational stressors related to suicide risk/suicidal ideation, while comparisons were not included in our examination. The outcomes were negative psychological outcomes (e.g., the presence of depression, anxiety, maladaptive coping strategies, and suicidal ideation/suicide risk).

Two independent reviewers read the titles and abstracts of the reports identified by the search strategy and performed initial screening; further selection was then made by analyzing the full text of the articles. The decision to include each article was made separately by the investigators; disagreements were resolved by discussion with a third reviewer. Authors discussed five papers and three were deleted. Data were extracted by two reviewers and inserted into a spreadsheet, including the country of origin of the study, the sociodemographic characteristics of the sample, the scale, and the instruments administered to the sample. Each paper was labeled with one or more main themes related to organizational factors in the workplace, and the main psychological outcomes were identified.

### 2.1. Inclusion Criteria

We included only articles in this review that provided insight into specific organizational stress issues and described organizational characteristics that may be related to suicide risk/suicidality and negative psychological outcomes. In addition, the selected articles included negative psychological outcomes, such as the presence of depression or anxiety symptoms, or outcomes related to workplace stress, such as emotional exhaustion or job satisfaction. We included only original articles in the review, although previous relevant review articles are discussed in the Section 1 and Section 4. Moreover, only articles written in English were included.

### 2.2. Exclusion Criteria

Reports, editorial articles, letters to the editor, non-peer-reviewed articles, articles written in a language other than English, and purely descriptive studies published at scientific conferences that did not include quantitative and qualitative conclusions were excluded from this review. In addition, we excluded any articles that described police officers’ mental health problems in suicide prevention in the general population without providing insight into workplace characteristics. In addition, we excluded articles that focused exclusively on the occupational and individual factors related to mental health problems and those in which organizational factors were not clearly identified. Finally, we excluded studies with samples of firefighters, prison police officers, special forces police officers, and other relevant groups.

## 3. Results

**Figure 1:**
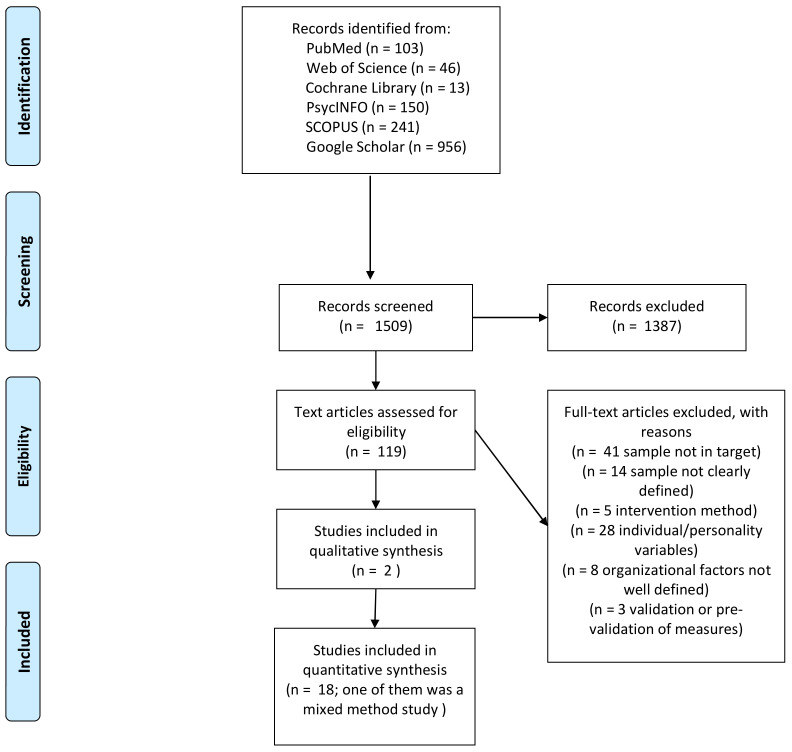
Article-selection algorithm (PRISMA) [32].

The literature search returned a total of 1509 results: 103 from PubMed; 46 from Web of Science; 13 from Cochrane Library; 150 from PsycINFO; 241 from SCOPUS; and 956 from Google Scholar. After the initial screening based on title and abstract, subsequent reading of the full texts, and removal of duplicates, a total of 20 papers were deemed suitable for inclusion in the review according to the inclusion and exclusion criteria. The selection process is described in Figure 1.

A total of 90% of the selected papers (18/20) were cross-sectional studies. Self-administered paper-and-pencil surveys were the most frequently used instruments to study the sample; the online method was used in 25% of the studies (5/20). Regarding the geographic origin of the included studies, European countries were the most represented (9/20, 45%), followed by those in North America (4/20, 20%), Central America (3/20, 15%), Asia (2/20, 14.3%), East Africa (1/20, 5%) and by Australia (1/20, 5%), 

Several organizational factors were analysed. Validated questionnaires used were the Police Stress Questionnaire (PSQ), Spielberger Police Stress Survey, Karasek’s Job Content Questionnaire, Effort-Reward Imbalance Questionnaire, Organizational Check-up Survey, Interpersonal Support Evaluation List-12, Social Relations Scale, Male Role Norms Scale. Ad hoc questionnaires were developed for some studies. Two studies followed a qualitative approach and conducted interviews, while one study used a mixed method. 

Regarding psychological dimensions, PTSD symptoms were assessed with the PTSD Checklist- Civilian version (PCL-C) and the Impact Event Scale-Revised (IES-R). Depression and anxiety symptoms were mainly tested with the Patient Health Questionnaire (PHQ), and anxiety symptoms were mainly tested with the Generalized Anxiety Disorder (GAD -2). Burnout was assessed mainly with the Maslach Burnout Inventory (MBI) scale and with the Copenhagen Burnout Inventory (CBI). Symptoms of distress and negative feelings were assessed with the Well-Being Index (WHO-5), the General Health Questionnaire (GHQ), the Salutogenetic Subjective Work Analysis Inventory, and the short version of the Symptom Check List 90 (BSI). In one study, suicidal ideation was tested with the Adult Suicide Ideation Questionnaire (ASIQ). For the sections related to organizational factors, ad hoc questionnaires were developed in some studies to capture psychological aspects as well. For other studies, a qualitative approach with interviews and open-ended questions was used. 

Analysis of the included articles revealed four main themes in organizational factors, the presence or absence of which could influence police officers’ well-being as follows:

1. Social support from the organization;
2. Leadership;
3. Organizational culture;
4. Bureaucracy.

The results for each theme are reported in the following paragraphs. A summary of the content of each item is provided in Table 1 and an overall summary of the results is provided in Table 1. To assess the quality and effectiveness of the studies, the checklist of Tufanaru et al. [33] was used. (see Appendix A, Table A1).

### 3.1. Social Support from Organization

Social support is the provision by an organization of psychological and material resources that can improve a person’s ability to cope with stress. In other words, social support can act as a stress buffer by eliminating or reducing the impact of stressful experiences by promoting fewer threatening interpretations of negative events and effective coping strategies. It can come in any form, as Cohen [34] mentions, and can create a sense of belonging and even help workers overcome obstacles. A total of nine studies (45%) [35,36,37,38,39,40,41,42,43] highlighting the importance of social support from organizations were included in this review. The absence or presence of social support from organizations was assessed in a cross-sectional study using a validated scale and an ad hoc questionnaire. In relation to the lack of organizational social support, this lack was found to have an impact on the increase of depressive symptoms, PTSD, burnout, and suicidality. The findings of Baek et al. [35] suggested that organizational social support is crucial in mitigating stress. The results of Baka’s [36] study showed that depressive symptoms increased among police officers who perceived low social support. Violanti et al. [37] showed that police officers who perceived low social support showed increased PTSD symptoms and used lower levels of active coping. In addition, Njiro et al. [38] found that police officers who perceived a lack of social support were at increased risk for depression and suicidality. Wray and Jarrett [39] also emphasized that limited opportunities for support can increase suicidal ideation and the risk of burnout. The presence of organizational social support has been identified as an important organizational factor that can increase officer well-being and reduce stress and strain. Tucker [38] found that perceived organizational support increased the possibility of contacting services for both individuals and stress intervention service users. In a study by Frank et al. [40], results indicated that organizational support reduced levels of job stress. Similarly, Santa Maria et al. [42] found that social support was negatively associated with psychological distress, with effects on depressive and anxiety symptoms. Kula [43] found that in the indirect causal effects of organizational and operational stress on job satisfaction, social support was a mediator. 

### 3.2. Organizational Culture

Organizational culture was defined by Schein [44] as “(1) a pattern of basic assumptions, (2) invented, discovered, or developed by a given group, (3) as it learns to cope with its problems of external adaptation and internal integration, (4) that has worked well enough to be considered valid and, therefore (5) is to be taught to new members as the (6) correct way to perceive, think, and feel in relation to those problems” (p. 7). In organizations, values and norms that constitute the culture could affect the perception of a good or bad climate or environment, of psychological strain [45], and could lead to failures in communication [46].

In this thematic review, five studies (25%) of those included highlight the importance of organizational culture [42,47,48,49,50]. Again, the studies show both positive and negative effects of a particular organizational culture. Santa Maria et al. [42] found that shared values were a workplace resource that was negatively associated with anxiety and depressive symptoms in police officers. In an interesting study by Gutschmidt and Vera [47], their results indicated that the type of organizational culture (e.g., hardworking cultures, team cultures) predicted both the maladaptive strategies used by police officers and their job satisfaction. In a study by Sitko-Dominik and Jakubowski [48], results showed a relationship between adherence to traditional masculinity norms (e.g., anti-femininity) and PTSD symptomatology (related to fear of losing emotional control). More specifically, their findings indicated that in male police officers, coping with stress could be negatively affected by their adherence to certain social role norms (e.g., hypermasculinity) acquired during the socialization process. The results of the qualitative research of Demou et al. [49] indicated that organizational culture is one of the main stressors contributing to male health problems among police officers, as values have an impact on stigma in seeking support. An interesting topic is the values that make up an organizational culture and the acceptance (or non-acceptance) of those values. In Rabe-Hemp’s [50] qualitative research, female officers described the option of being part of the organization and accepting the values (e.g., a masculine police culture oriented toward norms of violence, aggression, danger, solidarity, and courage) or of being part of the organization and not accepting the values, instead choosing more appropriate norms (e.g., exaggerating their unique skills).

### 3.3. Leadership

Leadership is the process of influencing others to understand and accept the decisions to be made and the actions to be taken, thereby facilitating individual and collective efforts to achieve a common goal. This process could have an impact on perceived workplace stress and work outcomes such as job satisfaction, turnover intention, and job performance [51]. 

A total of five studies (25%) highlighting the importance of leadership were included in this review [42,49,52,53,54]. In the study by Thompson et al. [52], results indicated that for female police officers, relationship conflict is a source of stress, particularly issues with poor leadership (e.g., lack of guidance and lack of positive feedback). Santa Maria et al. [42] found that a positive leadership climate, along with social support and shared values, was a work resource that was negatively associated with depression and anxiety symptoms in the police officers in their study. In a previous study by Russel [53], it was found that there was a lower perceived level of burnout in the group with a leader who exhibited high levels of transformational leadership. This type of leader is able to mitigate perceived burnout, especially when stress levels are low. When stress increases, the author suggests using a different leadership style (e.g., supportive leadership) that could mitigate perceived burnout. Leadership style could also influence perceived psychological health. Santa Maria et al. [54] specifically highlighted the role of a health-oriented leadership style on psychological and physical health: in their study, this leadership style was not only associated with improved well-being, but also with lower levels of burnout, depression, and physical complaints. The interviews conducted by Demou et al. [49] also revealed the importance of leading by example when it comes to police officers’ psychological health.

### 3.4. Bureaucracy

Bureaucracy represents the administrative apparatus for the exercise of legal power, i.e., a system of precise rules and regulations to be applied in a manner that tends to be impersonal and impartial through systematic, precise, and rational procedures [55]. Bureaucracy could be evaluated both instrumentally, on the basis of its expected contribution to realization, and deontologically, on the basis of the validity of the codes of conduct and the principles of reason, morality, organization, and governance on which bureaucracy as an institution is based [56]. A total of five studies (25%) that examined bureaucracy as an organizational stressor were included in this review [35,50,57,58,59,60]. This stressor could affect well-being, in particular it seems to increase somatization, anxiety, and depressive symptoms. Domingues and Machado [57] underlined that those administrative duties entail significant stress. The results of a study by Kim et al. [58] suggest that bureaucracy has a greater negative impact on male police officer groups (with consequences for somatization, anxiety, and depression) than on female police officer groups (with consequences for anxiety and depression). In addition, Jackman et al. [59] found in their mixed-methods study that increasing bureaucracy contributes to ’leaveism’, the practice of using extra time (e.g., on a vacation) to complete work tasks. This behavior is counterproductive because it affects the quality of leisure time and does not provide needed rest. Baek et al. [60] found that administrative tasks have a negative impact on health. Other studies have shown that bureaucracy does not have only negative effects. As Rabe-Hemp [50] showed, the amount of bureaucracy could have a positive impact on the promotion experience of female police officers: women employed in agencies with more sophisticated bureaucratic structures appear to have better performance. 

## 4. Discussion

The aim of this paper was to highlight the organizational factors that might influence the perceived discomfort of police officers. The studies included in the thematic review referred to specific factors, not the general term “organizational stressors” or “workplace stressors”. It is important to emphasize this distinction because most studies use the general term “organizational stressor” (referring to context) to distinguish from “operational stressor” (referring to content, such as exposure to danger, threat, and trauma) [18]. For our purposes, we selected the studies that examined specific organizational factors. The results indicate that organizational social support, organizational culture, leadership, and bureaucracy are the organizational factors associated with police officers’ perceived discomfort. These organizational factors have negative impacts on individuals, perceptions of stigma when contacting support services, anxiety and depressive symptoms, burnout, PTSD, suicidal thoughts, and other factors. All studies examined showed consistency: each reported result was cross-checked with the other studies and no inconsistencies were found. 

From an organizational perspective, there are some implications that could be useful for decreasing police officers’ perceived discomfort. For example, because organizational social support can transform maladaptive coping strategies into adaptive ones, it might be useful to provide an organizational support network [37]. More specifically, regular debriefings of stressful assignments could be offered to identify potential psychological distress and to provide support [42]. This practice implies that senior administrators and supervisors need to be trained to provide a supportive environment for police officers [35]. 

In terms of organizational culture, as Sitko-Dominik and Jakubowski [48] noted, it is important to revisit the social norms that characterize the police organization and to monitor the value of expressing or not expressing negative emotions such as worry and anxiety. For example, it might be useful to provide regular feedback to highlight behaviors that promote desirable values and norms. Feedback could also be useful in improving the psychological health and well-being of police officers [40,43,49]. In agreement with Gutschmidt and Vera [47], it is also necessary to reconsider the composition of work groups and the balance of age and gender in teamwork. This could make it possible to reduce barriers to achieving high ranks and could ensure equal opportunities for men and women in the police organization [50].

In conjunction with organizational culture and the social support from the organization, leadership is an organizational factor that could reduce symptoms of emotional exhaustion, depression, and anxiety among police officers [42]. The style chosen by leaders could promote health-oriented behaviors, both physical and psychological. Therefore, supervisor training is important to create an environment where values and norms accommodate both the expression of negative emotions and a supportive climate. In particular, transformational leadership could be a style that fits well in police organizations and allows the promotion of well-being to be implemented in these organizations [53,54]. Another organizational factor that emerged from this report is bureaucracy, i.e., insufficient budgets, insufficient staff, inadequate policies and procedures, too much bureaucracy, and other related factors. As with the previous organizational factors, bureaucracy exhibits both positive and negative aspects. For example, it enables effective and efficient management systems and the control of applied procedures. However, in police organizations, it is difficult to centralize all operations and limit the discretion of tasks, especially those of frontline officers. Autonomy in the workplace is essential for dealing with everyday situations that are outside the routine, such as the work of police officers. To counter bureaucracy, it may be useful to redesign the job to reduce the workload and staffing shortages [42,57,58].

Overall, data from this thematic review indicate the need to develop tailored interventions aimed at alleviating stress by training supervisors, rewriting procedures, examining psychological health symptoms, and offering support in seeking professional help [38,41,59]. All actions should be taken and promoted at the organizational level to ensure the effectiveness of the intervention. Interventions to address organizational stressors could be helpful in improving the well-being of police officers. For example, by allowing them to talk about their discomfort and by promoting greater transparency in decision-making processes and personnel management. Measures to address organizational and operational stressors would improve the quality of patrol officers’ work lives and have a positive impact on community service.

### Strengths and Limitations

This review has several strengths that need to be highlighted. First, the literature was specifically searched for organizational factors that might impact police officers’ discomfort, which provided an overview of psychological health outcomes from an organizational perspective. Therefore, we focused our study on the negative factors because they are the best studied [61]. However, the results of this study open interesting perspectives for studies dealing with job satisfaction, work engagement, well-being, and happiness in the police officers’ organizations. The results provide an opportunity to reflect on which organizational aspects are undesirable. However, aspects that can serve as protective and their effects on police officers were not examined in this study. Further research could focus on the positive aspects and identify which aspects should be considered as having a moderating effect on the perception of organizational stressors. In addition, the selection of articles focused on papers that provided insight into working conditions and made some connections between psychological outcomes and specific psychosocial factors in the workplace. Although a qualitative approach was taken in this review, this is consistent with the PRISMA statement, which lends more weight to the findings. Finally, unlike previous research, this study also focused on police officers and recognized organizational characteristics that impact psychological health in their daily work. However, some limitations should be noted. Most of the selected studies were cross-sectional in nature, which limits the power of the results and the possibility of making causal conclusions. In addition, the data were collected using different methods and in different countries, which may have influenced the results, especially given the heterogeneity of police organizations in the world. Another issue concerns the model, scales, and instruments used to screen participants and the appropriateness of sample selection. For example, further analysis of the literature could examine the specific Job Demand Resource Model, one of the most commonly used models to study work-related stress. Only papers written in English were considered, so many interesting studies were excluded. In addition, maladaptive coping strategies were considered an outcome rather than a moderator. All negative psychological outcomes fall under the hedonic term; we did not consider eudaimonic (also negative) outcomes. Further analysis of the literature could consider eudaimonic outcomes to better understand the phenomenon. Some specific organizational intervention opportunities were identified that could protect and promote workers’ psychological health. However, we did not discuss these interventions, even if they could provide a practical guide for future reviews of the literature and serve as a possible stimulus for findings. Even more, we focused on the specific population of police officers. Other reviews could be conducted for other similar occupational groups such as firefighters, correctional officers, and other related groups.

## 5. Conclusions

The importance of improving working conditions for the health and psychological health of police officers creates added value in terms of organizational performance and the quality of life of those in the organization. Protecting the psychological health of police officers could have a positive impact not only on the private lives of employees but also on the quality of the relations between police officers and the users of police services, which, in turn, may lead to an improvement in the services offered to citizens. It is therefore desirable that all police organizations become aware of the importance of psychosocial risk assessment for not only the protection of police officers’ health but also for the optimization of their responses to citizens’ requests. 

## Figures and Tables

**Table 1:** Summary of main findings and implications.

	Major Findings	Implications
Social Support From Organization	- the lack of social support from supervisor is the major source of stress in police officers; - mediates the indirect causal effect of both organizational and operational stress on job satisfaction; - in response to the lack of support, police officers’ PTSD symptoms increase; - the lack of social support from the organization increases the risks of depression and of suicidality.	- organizational support is crucial in helping officers deal with stressful and traumatic events; - police officers who perceive organizational support are more willing to use stress intervention services; - management training for senior staff could develop a supportive environment among co-workers.
Organizational Culture	- determines the strategies for coping with stressful situations; - diligence culture and team culture seem to be protective factors against maladaptive coping; - norms and values oriented to masculinity provoke the greatest risk of resistance, harassment, and isolation, particularly in female police officers.	- for the development of long-lasting police-specific stress prevention, treatments and initiatives, a change in organizational culture is essential: - a culture of wellness helps to fight the stigma of seeking support; - could be useful to rethink traditional practices in selection, training, and performance evaluation.
Leadership	- a negative leadership climate constitutes a job demand; - negative leadership style is associated with the experience of psychological strain, emotional exhaustion, and depression and anxiety symptoms.	- leadership could be addressed as a valuable resource with regard to organizational health by educating police leaders in the principles of health-oriented leadership and by promoting health-oriented awareness; - authentic leadership, participation options, supportive management, and regular feedback help to improve cohesion in teamwork.
Bureaucracy	- this is an important source of stress for police officers; - increases somatization, anxiety, and depression; - affects perceived well-being.	- management has to redesign the work, increasing flexibility, autonomy, and discretion for arranging work. - could be useful to offer a different shift system with stipulated working hours; - necessary to rewrite tasks and work routines; - the need to increase the number of police officers on staff.

## Appendix A

**Table A1:** Checklist to assess the quality of the study included in the narrative review.

References	Q1	Q2	Q3	Q4	Q5	Q6	Q7	Q8	Q9
Baek et al., 2021 [35]	Yes	No	No	No	No	No	No	Yes	Yes
Baka et al., 2020 [36]	Yes	No	No	No	No	No	No	Yes	Yes
Violanti et al., 2018 [37]	Yes	No	No	No	No	No	No	Yes	Yes
Niiro et al., 2021 [38]	Yes	No	No	No	No	No	No	Yes	Yes
Wray and Jarrett, 2019 [39]	Yes	No	No	No	No	No	No	Yes	Yes
Tucker, 2015 [40]	Yes	No	No	No	No	No	No	Yes	Yes
Frank et al., 2017 [41]	Yes	No	No	No	No	No	No	Yes	Yes
Santa Maria et al., 2018 [42]	Yes	No	No	No	No	No	No	Yes	Yes
Kula, 2017 [43]	Yes	No	No	No	No	No	No	Yes	Yes
Gutschmidt and Vera, 2021 [47]	Yes	No	No	No	No	No	No	Yes	Yes
Sitko-Dominik and Jakubowski, 2021 [48]	Yes	No	No	No	No	No	No	Yes	Yes
Demou et al., 2020 [49]	Yes	No	No	No	No	No	No	Yes	Yes
Rabe-Hemp, 2008 [50]	Yes	No	No	No	No	No	No	Yes	Yes
Thompson et al., 2006 [52]	Yes	No	No	No	No	No	No	Yes	Yes
Russell, 2014 [53]	Yes	No	No	No	No	No	No	Yes	Yes
Santa Maria et al., 2019 [54]	Yes	No	No	No	No	No	No	Yes	Yes
Domingues and Machado, 2017 [57]	Yes	No	No	No	No	No	No	Yes	Yes
Kim et al., 2016 [58]	Yes	No	No	No	No	No	No	Yes	Yes
Jackman et al., 2021 [59]	Yes	No	No	No	No	No	Yes	Yes	Yes
Baek et al., 2021 [60]	Yes	No	No	No	No	No	No	Yes	Yes

Note. Q1: Is it clear in the study what the ‘cause’ is and what the ‘effect’ is? (i.e., there is no confusion about which variable comes first); Q2: Were the participants included in any similar comparisons?; Q3: Were the participants included in any comparisons receiving similar treatment/care other than the exposure or intervention of interest?; Q4: Was there a control group?; Q5: Were there multiple measurements of the outcome both pre and post the intervention/exposure?; Q6: Was follow-up completed and if not, were the differences between the groups in terms of their follow-up adequately described and analyzed?; Q7: Were the outcomes of participants included in any comparisons measured in the same way?; Q8: Were outcomes measured in a reliable way?; Q9: Was appropriate statistical analysis used? Possible answers: Yes, No, Unclear (U), or Not Applicable (N/A).
